# Authority, trust, and healthcare communication in a religious minority: the case of the Haredi community in Israel during COVID-19

**DOI:** 10.1186/s12889-026-27338-9

**Published:** 2026-04-30

**Authors:** Sara Zalcberg, Yanyan Chen, Sima Zalcberg-Block

**Affiliations:** 1 Department of Psychology, Jerusalem Multidisciplinary College, Jerusalem, Israel; 2 SDU-TAU Joint Institute for Jewish ans Israel Studies, Tel Aviv University, Tel Aviv, Israel; 3 The Center for Judaic and Inter-religious Studies, The School of Philosophy and Social Development, Shandong University, Shandong, China; 4 School of Social Work, Ariel University, Ariel, Israel

**Keywords:** Haredi community, Religious minorities, Health equity, Health communication, Communication ecology, Trust, Healthcare barriers, COVID-19

## Abstract

**Background:**

Religious minority communities face distinctive healthcare barriers during public health crises, yet the communicative mechanisms underlying these barriers remain underexplored. Applying Communication Ecology Theory, this study examines barriers to healthcare access in Israel’s Haredi (ultra-Orthodox) community during the COVID-19 pandemic, conceptualizing healthcare inequality as a function of bounded communication ecologies rather than individual non-compliance, with implications for health equity in culturally and religiously diverse societies.

**Methods:**

A qualitative study was conducted using semi-structured interviews with 30 Haredi community members and analysis of 20 pashkevils (religious street posters) circulated during the pandemic. The Haredi community experienced infection and mortality rates substantially higher than the national average during COVID-19. Both data sources were analyzed thematically, guided by Communication Ecology Theory.

**Results:**

Three interconnected barriers emerged: (1) internal versus external-secular communication—reliance on community channels that conveyed partial or misleading health information; (2) rabbinic versus professional authority—prioritization of rabbinic rulings over medical guidance; and (3) religious versus scientific interpretation—viewing the pandemic as divine punishment requiring spiritual rather than medical responses.

**Conclusions:**

Healthcare barriers in the Haredi community reflect bounded communication ecologies where information legitimacy depends on alignment with religious authority and theological worldviews. Effective public health engagement requires culturally adapted strategies that operate within existing communication systems, collaborate with rabbinic leadership, and address both informational and structural barriers, thereby advancing health equity in religious minority populations. These findings are relevant for understanding healthcare barriers among religious minorities worldwide.

**Supplementary Information:**

The online version contains supplementary material available at 10.1186/s12889-026-27338-9.

## Background

Healthcare barriers are not merely structural obstacles but communicative processes shaped by authority hierarchies, trust networks, and culturally bounded systems of meaning. In the context of this study, healthcare barriers refer primarily to barriers in accessing and responding to public health information and services during the COVID-19 pandemic, including preventive measures (e.g., vaccination, social distancing), interaction with healthcare providers, and engagement with official health guidelines.

Religious minority communities around the world face distinctive barriers to healthcare that reflect broader issues of cultural autonomy, authority structures, and social isolation [1–3]. These challenges become particularly visible during health emergencies, when public health policies intersect with deeply rooted religious values and local systems of trust. The COVID-19 pandemic highlighted how differences in communication, leadership, and theology can significantly impact compliance with health directives, vaccination behavior, and access to accurate information [4, 5]. These dynamics have direct implications for health equity, as unequal access to culturally legitimate information and trusted authority structures may systematically disadvantage religious minority communities during public health crises.

In Israel, the Haredi (ultra-Orthodox) Jewish community, a conservative religious minority, represents a striking example of this dynamic. The Haredi community experienced infection and mortality rates significantly higher than the national average [6]. By June 2020, residents of Bnei Brak, a predominantly Haredi city, accounted for 15% of COVID-19 deaths while representing just 2% of the national population. At the pandemic’s peak, about 70% of infections occurred among Haredim [7], and by mid-October 2020, mortality rates within the community were four times higher than those in the general population [8].

Understanding this gap requires moving beyond simple models of “non-compliance” and considering the broader ecology of religious authority, internal communication, and theological meaning-making that structures everyday life in insular faith communities.

Accordingly, this study examines the specific barriers that shaped healthcare access during the COVID-19 pandemic, conceptualizing healthcare inequality as a function of bounded communication ecologies rather than individual non-compliance. By analyzing interviews and communal media, the study seeks to map the key barriers to healthcare in the Haredi context and to illuminate how these barriers reflect the broader dynamics of authority, trust, and information flow in religious enclave societies. This study advances health communication scholarship by demonstrating how communicative legitimacy—rather than mere information availability—structures healthcare access in religious enclave societies, thereby shaping broader patterns of health equity.

This study advances existing scholarship in several ways. First, in contrast to prior research on COVID-19 in the Haredi community, which has focused primarily on compliance, vaccination behavior, and perceptions of official guidelines, the present study shifts attention to communicative processes as key mechanisms shaping healthcare barriers. Rather than examining behavioral outcomes alone, it focuses on the underlying communicative structures that produce and sustain these outcomes.

Second, the study contributes methodologically by integrating two complementary data sources—semi-structured interviews and pashkevils (religious street posters [9]). The combined analysis of these sources enables a more comprehensive understanding of how private experiences and publicly circulating communal discourses interact within the communication ecology. This approach captures both lived interpretations and normative messages, thereby illuminating the dynamic relationship between individual meaning-making and collective communication structures. Third, the study extends Communication Ecology Theory by demonstrating how theological worldviews function as an additional layer shaping information legitimacy within bounded communication systems.

Beyond these empirical and methodological contributions, the study offers a conceptual contribution by reframing healthcare inequality in religious minority contexts as a function of communicative legitimacy rather than information access alone. While prior research has largely interpreted gaps in compliance through the lenses of trust, attitudes, or structural barriers, this study conceptualizes health behavior as emerging from bounded communication ecologies in which authority, theology, and internal media jointly define what counts as valid knowledge. This perspective shifts the analytical focus from individual decision-making to the sociocultural production of legitimacy, thereby extending Communication Ecology Theory into the domain of religiously embedded health communication.

### Religious minorities and health behaviors in health crises

Research on religious minorities consistently highlights a dual structure of barriers to healthcare—external and internal. Externally, religious groups often encounter discrimination, lack of culturally adapted services, and language or bureaucratic challenges that limit access to medical care [10]. Internally, religious norms, gendered expectations, and theological interpretations shape attitudes toward illness and treatment [11]. These perspectives influence whether believers seek medical assistance, adhere to preventive measures, or prioritize prayer and ritual purity as mechanisms of healing.

Religious leaders play a decisive role within this structure. In many faith communities, clerics or spiritual authorities act as moral interpreters of medical guidance, determining which practices align with religious law and which are perceived as threatening to faith [12, 13]. Their approval or disapproval can significantly influence communal attitudes toward vaccination, quarantine, and other interventions [3]. During public health crises, such authority becomes both a potential bridge and a barrier. When religious leaders collaborate with medical institutions, they can effectively translate public health messages into culturally legitimate language [14, 15]. However, when trust in secular systems is low or when religious autonomy is perceived as under attack, religious leaders may promote resistance, framing state health policies as violations of religious freedom [16].

The COVID-19 pandemic made these dynamics acutely visible across the globe. In Europe and North America, some religious groups—such as Evangelical churches, Orthodox Christian communities, and Muslim congregations—initially resisted lockdowns, viewing restrictions on collective worship as spiritual oppression [5, 17]. Simultaneously, other religious organizations reframed compliance as a moral and religious duty and a means of caring for the vulnerable [18]. These contrasting responses underscore how the moral framing of health directives—whether as an act of faith or as a threat to it—determines collective behavior.

Insular religious groups, including the Amish in the United States, exemplify how closed communication systems reinforce these patterns. Lacking integration with mainstream media, they rely on community-specific information networks—religious newsletters, local radio, or word-of-mouth communication—that both strengthen group cohesion and limit exposure to scientific discourse [19]. As a result, misinformation can circulate unchecked, while state messaging often fails to penetrate [5]. Thus, the health behavior of religious minorities during crises is best understood not as irrational resistance but as a rational response within distinct moral and communicative systems—one that can only be fully grasped through contextualized, culture-sensitive perspectives.

### The Haredi community in Israel

The Haredi community in Israel provides an especially illustrative case of how religious identity, communication patterns, and authority structures interact to shape health behavior. The Haredi population is characterized by a distinctly young demographic profile—about 60% are under the age of 20, compared to 35% in the general Israeli population. Its socioeconomic status is relatively low [6], and it experiences high population density and crowded living conditions [20]. These structural conditions intensified the spread of COVID-19 in Haredi areas, amplifying the community’s epidemiological vulnerability [21].

The Haredi population is divided into three major streams: Hasidim, organized around charismatic rebbes and emphasizing spiritual devotion; the Lithuanian stream, which prioritizes Torah study and a relatively pragmatic approach to modernity; and the Sephardi stream, which has adopted a lifestyle similar to the Lithuanian model while maintaining Middle Eastern traditions [22, 23]. Another key distinction is between mainstream Haredim, who maintain pragmatic relations with the State of Israel, and extremist factions that reject its legitimacy altogether [24, 25]. While mainstream groups generally respect state laws and cooperate with governmental institutions, extremist circles actively resist state authority [26] and have been involved in protests and violent confrontations with police during lockdown enforcement [7, 9].Despite the internal diversity of the Haredi population, many Haredi groups are characterized by strong adherence to Halacha and by the significant influence of rabbinic authority, although the extent of such influence varies across subgroups [22]. All subgroups are integrated into a tightly knit social system that emphasizes men’s commitment to Torah study, and stringent modesty norms [27, 28], particularly regarding women’s clothing [29, 30]. The community maintains an autonomous infrastructure of education, welfare, and media that minimizes contact with secular institutions.

Despite ongoing isolationist tendencies, the past two decades have seen a gradual process of partial integration into Israeli society, higher education, and the labor market [31]. This trend has led to greater cooperation with state institutions, including the healthcare system, even among groups once considered zealously separatist [24].

Nevertheless, many segments of the Haredi community still rely primarily on internal communication channels—particularly street posters known as *pashkevils* —that serve as tools for disseminating religious and social messages. *Pashkevils* traditionally function as both instruments of rabbinic authority and platforms for communal debate [32, 33]. Even as digital technologies increasingly penetrate Haredi life [6], these pashkevils continue to play a symbolic and communicative role —especially during the COVID-19 pandemic, when traditional media channels within the community became central to meaning-making and information exchange [34].

### Health, illness, and the COVID-19 pandemic in the Haredi community

Health and illness in the Haredi worldview are framed within a religious context. Maintaining one’s health is perceived as a religious obligation (Deuteronomy 4:15)—interpreted as a directive to protect bodily well-being. Nevertheless, empirical studies show low engagement in health-promoting behaviors, consistent with the community’s lower socioeconomic status [35, 36].

Paradoxically, Haredim enjoy relatively high overall health and life expectancy, attributed to strong social capital, cohesive family structures, mutual aid, and faith-based resilience [37, 38]. Rabbinic authority plays an important role in shaping health-related behavior in many Haredi communities; however, its influence varies across subgroups and contexts, and patterns of compliance during the COVID-19 pandemic were neither uniform nor static over time. Structural factors, including socioeconomic conditions and access to resources, also played a significant role in shaping responses [39, 40].

A growing body of research has examined how the Haredi community navigated the COVID-19 crisis. Scholars have explored patterns of compliance and defiance toward state regulations [7], the shaping role of rabbinic authority [39], and public trust and vaccination attitudes [14, 41]. Collectively, these works underscore the complexity of health communication in religious enclave societies.

Yet, despite their contributions, most analyses remain external—focusing on compliance rates, epidemiological data, or state-community relations—leaving a limited understanding of the internal mechanisms that constructed healthcare barriers within Haredi life. Accordingly, this study examines the concrete barriers that shaped healthcare access in the Haredi community during the COVID-19 pandemic, revealing how structural, religious, and communicative factors interacted to hinder the transmission and acceptance of public health information.

### Theoretical framework: communication ecology theory

Communication Ecology Theory [42, 43] provides a conceptual framework for understanding how communities construct meaning and transmit information through interdependent networks of people, technologies, and institutions. More recent applications of the theory emphasize its value in analyzing health communication and crisis response, illustrating how communication resources cluster and interact during public emergencies [44, 45].

A “communication ecology” consists of overlapping layers—individual, social, and institutional—that collectively shape how information flows and how legitimacy is assigned to messages. Within religious communities, these ecologies are bounded by theological worldviews and authority hierarchies that define what constitutes trustworthy knowledge.

Applying this framework to the Haredi case highlights why mainstream health communication frequently fails in insular societies. Messages originating from secular authorities must pass through communal gatekeepers—rabbis, educators, and localized media—who reinterpret them according to internal religious logics. This filtering process can transform, delay, or even block critical health information. Communication Ecology Theory thus shifts analytical attention from the content of messages to the relational and cultural systems through which meaning is negotiated.

Ultimately, this approach underscores that health communication is not merely the transmission of facts but the construction of shared legitimacy within culturally specific systems of meaning. For the Haredi community—and for religious minorities more broadly—understanding and engaging with this communication ecology is essential for effective crisis management and long-term trust-building.

## Methods

This study employed a qualitative research design to generate an in-depth understanding of the healthcare barriers faced by members of the Haredi community in Israel during the COVID-19 pandemic. A qualitative approach was selected because it enables exploration of meaning-making processes, authority structures, and communication practices within culturally bounded communities—phenomena not easily captured through quantitative measures [46].

Data were collected between March 2020 and November 2021, a period encompassing the major waves of the pandemic in Israel, thereby allowing examination of evolving perceptions and responses. The study drew on two complementary data sources to enable triangulation: (1) semi-structured, in-depth interviews with members of the Haredi community, and (2) pashkevils - street posters circulated in Haredi neighborhoods. Integrating these sources made it possible to compare private narratives with publicly disseminated communal messaging, thereby strengthening analytic credibility.

### In-depth interviews

#### Recruitment

Purposive sampling was employed to recruit participants capable of providing rich, experience-based insight into healthcare decision-making and information practices during the pandemic [47]. Initial participants were identified through the authors’ contacts established during prior research within Haredi society. These initial contacts were purposively selected to ensure variation across key subgroups (Hasidic, Lithuanian, and Sephardi streams, as well as more mainstream and more insular factions), and to include participants with different occupational and social backgrounds. These individuals subsequently facilitated introductions to additional participants via referral chains (snowball sampling), a strategy particularly appropriate when studying socially bounded populations characterized by high levels of internal trust.

Snowball sampling was then used to expand the sample through participants’ social networks. While this approach facilitated access to a socially bounded population, it may have led to partial clustering of participants within specific social circles, potentially over-representing particular subgroups or perspectives.

In addition, the recruitment process resulted in a sample composed exclusively of married adults aged 25 and above. This reflects both the structure of social networks through which recruitment occurred and cultural norms within the community. However, it may have excluded the perspectives of younger and unmarried individuals, whose experiences and information practices may differ. These sampling characteristics should therefore be considered when interpreting the findings, as they may have influenced the patterns identified.

Ultimately, the study sample consisted of 30 participants. Data saturation was assessed iteratively throughout the data collection and analysis process. After approximately 25 interviews, no substantially new themes or conceptual insights emerged, indicating thematic redundancy. Five additional interviews were conducted to confirm saturation and ensure the robustness of the thematic structure.

Participants included 18 men and 12 women, aged between 25 and 65 years. To protect participant anonymity, ages are presented in Table 1 as age ranges rather than exact ages. All participants were married and, on average, had six children. Twenty-one participants identified with the mainstream Haredi groups, while nine were affiliated with more insular or extremist Haredi factions. All participants lived in Haredi neighborhoods, some of which were in predominantly Haredi cities. In terms of occupation, participants included eight working in education, six in business, finance, or administrative roles, five in healthcare and social services, eight men engaged in full-time Torah study (Kollel), and four women who were homemakers (Table 1).

**Table 1:** Participants’ demographic characteristics (*n* = 30)

Participant No.	Gender	Age	Stream	Sector	Employment
1	Female	25–34	Sephardi	Mainstream	Healthcare/ Services
2	Male	45–54	Hasidic	Extremist	Business/Finance
3	Male	35–44	Hasidic	Mainstream	Full-time Torah study
4	Male	45–54	Litvak	Mainstream	Education
5	Male	35–44	Litvak	Mainstream	Full-time Torah study
6	Female	55–65	Hasidic	Mainstream	Homemaker
7	Male	55–65	Litvak	Extremist	Full-time Torah study
8	Female	25–34	Hasidic	Extremist	Homemaker
9	Male	25–34	Litvak	Mainstream	Full-time Torah study
10	Female	45–54	Hasidic	Extremist	Education
11	Female	35–44	Litvak	Mainstream	Education
12	Male	35–44	Litvak	Mainstream	Business/Finance
13	Male	35–44	Litvak	Mainstream	Business/Finance
14	Female	35–44	Litvak	Extremist	Education
15	Male	35–44	Sephardi	Mainstream	Full-time Torah study
16	Male	35–44	Hasidic	Mainstream	Education
17	Male	35–44	Litvak	Mainstream	Healthcare/ Services
18	Female	35–44	Hasidic	Extremist	homemaker
19	Female	35–44	Hasidic	Mainstream	Full-time Torah study
20	Female	45–54	Sephardi	Mainstream	Healthcare/ Services
21	Male	55–65	Hasidic	Extremist	Full-time Torah study
22	Male	35–44	Litvak	Mainstream	Education
23	Female	25–34	Sephardi	Mainstream	Business/Finance
24	Male	35–44	Litvak	Mainstream	Business/Finance
25	Male	35–44	Sephardi	Mainstream	Healthcare/ Services
26	Male	25–34	Hasidic	Extremist	Full-time Torah study
27	Male	25–34	Hasidic	Mainstream	Education
28	Female	25–34	Litvak	Mainstream	Education
29	Female	25–34	Sephardi	Mainstream	Business/Finance
30	Male	25–34	Hasidic	Extremist	Healthcare/ Services

#### Interview procedure

Semi-structured interviews were conducted using an interview guide that addressed several main topics, including personal and family experiences during the pandemic, the difficulties and challenges encountered, the role of religious authorities in health-related decision-making, and communal responses to governmental and public health measures.

The interview guide consisted primarily of open-ended questions designed to elicit narrative accounts while minimizing interviewer direction. The guide remained adaptable throughout the interviews, allowing participants to raise issues they considered significant. Each interview lasted between 60 and 90 min and was conducted in Hebrew—20 by telephone and 10 via Zoom—to comply with COVID-19 safety guidelines.

The first and last authors conducted all interviews. Both have extensive experience in qualitative research and prior fieldwork in Haredi communities [48–50]. Their familiarity with the cultural context facilitated rapport and supported nuanced interpretation. To mitigate potential bias associated with this proximity, analytic decisions were discussed among the research team throughout the study. With participants’ consent, all interviews were audio-recorded and transcribed verbatim.

### Pashkvils

#### Data collection and documentation

Pashkevils were identified using a linguistic landscape (LL) approach [51], utilizing systematic environmental scanning to capture naturally occurring public communications within community settings [52]. Photographic documentation was conducted between March 2020 and March 2021 in major Haredi population centers in Israel, including Jerusalem, Beit Shemesh, Bnei Brak, and Beitar Illit. These locations were selected for their demographic diversity and representation of multiple Haredi subgroups, ranging from moderate to more insular communities.

Local research assistants conducted field documentation two to three times per week, targeting central streets, neighborhood gathering points, synagogue vicinities, and commercial areas where pashkevils are commonly displayed. For each pashkevil, the following metadata were recorded: date of photograph, geographic location (city and neighborhood), and approximate posting date. While duplicates across multiple locations were documented to track prevalence, they were treated analytically as recurrent messages rather than separate items.

#### Sampling and selection criteria

From the broader photographic archive, a final sample of 20 pashkevils was selected for analysis using purposive sampling. This approach aligns with qualitative strategies that prioritize information-rich cases capable of illuminating the phenomenon under investigation. Selection was guided by the study’s objective to learn about healthcare barriers, rather than achieving representational balance.

To be included in the final sample, pashkevils had to meet the following thematic criteria: relate to explicit or implicit discouragement of compliance with public health measures or medical guidance. Pashkevils that did not reflect this criterion were excluded. Within the relevant thematic scope, preference was given to pashkevils that were widely visible, recurrent across locations, and textually rich.

The final sample of 20 pashkevils was determined based on principles of information richness and thematic sufficiency rather than numerical representativeness. Sampling continued until recurring discursive patterns were identified across multiple posters, and no substantially new themes emerged from additional materials. This approach is consistent with qualitative research practices that prioritize depth and variation over sample size alone.

### Data analysis

Analysis was conducted in Hebrew to preserve linguistic nuance; selected quotations were subsequently translated into English for reporting. All interviews were transcribed, de-identified, and analyzed alongside the pashkevils using thematic analysis.

The analysis of pashkevils focused primarily on textual content, which was coded thematically alongside interview data. Visual elements (e.g., typography, layout, emphasis, and use of symbols) were considered as supporting indicators of message salience and authority, but were not systematically coded as separate analytic categories.

Authorship was typically anonymous or attributed to collective religious authority, and was therefore interpreted as part of the communicative structure rather than as an individual characteristic. Where identifiable, references to rabbinic figures or groups were treated as indicators of perceived authority and legitimacy.

Duplicate pashkevils documented across multiple locations were not treated as separate units of analysis, but rather as indicators of message diffusion and prominence within the communication ecology. While the pashkevils were collected from the same communities in which participants resided, the study does not assume uniform exposure among interviewees. Instead, pashkevils were analyzed as part of the broader communicative environment shaping available discourses, rather than as direct measures of individual-level information consumption.

The analysis was conducted manually using an iterative and systematic coding process, without the use of dedicated qualitative analysis software. Interview transcripts and pashkevils were analyzed using thematic analysis, as outlined by Braun and Clarke [53], according to the following steps: (1) familiarization with the data through repeated reading of transcripts and pashkevils to immerse in participants’ narratives and discursive patterns; (2) initial coding, where meaning units were identified and assigned descriptive and interpretive codes; (3) organizing codes into potential themes by comparing and collating them into preliminary categories reflecting recurring patterns; (4) theme development, involving the clustering of code families into candidate themes while ensuring internal coherence and external distinction; (5) theme refinement, where candidate themes were iteratively reviewed against the dataset to adjust boundaries, merge or separate subthemes, and sharpen definitions; and (6) defining final themes and mapping their relationships.

The first and last authors, both native Hebrew speakers, independently conducted the initial coding. Inter-coder agreement was calculated as percentage agreement across a subset of transcripts, yielding a high level of agreement (approximately 85%). Discrepancies were discussed and resolved through consensus in collaboration with the research team.

### Quality assurance

The study employed multiple strategies to ensure research quality [46, 54]. Credibility was established by including participants from diverse Haredi subgroups—ranging from moderate to conservative communities—to capture a wide range of perspectives within Haredi society during the COVID-19 pandemic. In-depth, semi-structured interviews encouraged open and authentic dialogue, while iterative review of data collection and analysis enhanced consistency. Furthermore, the selected pashkevils were treated as public cultural texts reflecting communal norms, authority structures, and meaning-making processes. When analyzed alongside interview narratives, they enabled triangulation between private accounts and publicly disseminated messages, thereby strengthening the credibility of the findings. Peer debriefing and the incorporation of colleagues’ feedback further supported the trustworthiness of the results.

To strengthen confirmability, the authors engaged in systematic reflexivity. Because qualitative research is inherently shaped by researcher positionality, this reflexive practice was maintained throughout the study. The research team includes scholars with backgrounds in psychology and social work and prior experience studying Haredi society. While such familiarity enhanced contextual sensitivity [55, 56], analytic awareness was preserved through documentation of assumptions, critical examination of preliminary interpretations in team discussions, and systematic grounding of claims in the data.

The researchers’ positionality also influenced data collection. The Israeli Jewish researchers, who are familiar with Haredi culture, facilitated access and rapport with participants, enabling open and nuanced discussions. At the same time, this insider proximity required ongoing reflexive attention to avoid normalization of culturally embedded assumptions. In contrast, the Chinese non-Jewish co-author contributed an outsider perspective, challenging taken-for-granted interpretations and supporting critical examination of culturally specific meanings, thereby enhancing reflexive balance within the research team.

Potential power dynamics during interviews were also considered. Given the hierarchical and gendered structure of Haredi society, participants may have perceived the researchers as authority figures or external evaluators. Efforts were made to minimize such dynamics by conducting interviews in a respectful, non-judgmental manner, allowing participants to guide the conversation, and emphasizing their autonomy in sharing experiences.

In this context, it should be noted that two members of the research team are Israeli Jewish scholars, while the third is a Chinese Christian scholar specializing in the study of religion and Haredi society. Reflexivity was maintained through ongoing analytic discussions among the research team. Transferability was supported through comprehensive contextual descriptions, detailed presentation of participant characteristics, and transparent documentation of the research process, enabling readers to assess the relevance of the findings to other Haredi contexts [57].

### Ethical considerations

The study received ethical approval from the Ethics Committee of the university under whose auspices the research was conducted. All participants provided informed consent after receiving detailed information regarding the study’s purpose and their rights, including the voluntary nature of their participation and the right to withdraw at any stage. Confidentiality was ensured through data de-identification, the use of generic identifiers (e.g., Participant 1), and secure storage of recordings on the university’s protected cloud server.

Pashkevils were treated as publicly available materials displayed in open community spaces. Their documentation did not involve interaction with individuals and was considered to pose minimal ethical risk. Public display was therefore understood as implied consent for observation and documentation of these materials. This procedure was included in and approved as part of the study’s overall ethics protocol.

## Results

Analysis of the interviews and pashkevils revealed three central themes that illuminate major health barriers among the Haredi population in Israel during the COVID-19 pandemic: first, internal versus external-secular communication; second, rabbinic versus professional authority; and third, religious versus scientific interpretation.

### Internal versus external-secular communication

Participants’ accounts revealed that due to their religious avoidance of “external-secular” media, many were not exposed to Ministry of Health (MOH) information and guidelines. Instead, they received most pandemic information through intra-community channels—primarily word of mouth, Nayes (Yiddish news) hotlines (telephone news channels serving the Haredi population), and pashkevils. These sources sometimes provided partial or contradictory information that undermined participants’ ability to understand the situation’s severity and respond appropriately. One participant described the consequences of avoiding “secular” media: “My husband and I had a high fever, and my neighbor told me about a serious epidemic, and we understood we had probably gotten infected. Until then, we hadn’t heard anything” (Participant 1).

Some participants reported that in the first weeks of the pandemic, they received only rudimentary information through Nayes hotlines. These channels provide updates independent of institutionalized Haredi press, several times daily, primarily for those avoiding television and the internet. As one participant recounted:

Since we avoid television and internet, we had no information regarding required or forbidden behaviors. Only in the evening, I heard on the “Nayes” we should avoid physical contact because of a contagious epidemic. So we didn’t shake hands. That’s all we knew (Participant 2).

This participant, along with others, indicated that Nayes hotlines provided extremely partial information, especially early in the pandemic, omitting critical MOH guidelines. Adherence to MOH guidelines could have significantly reduced infection rates in the Haredi sector and saved lives.

The gap between vital health information disseminated through secular media and pandemic response, and that received through intra-community channels, thus constituted a significant health barrier. This barrier was particularly evident regarding COVID-19 vaccines. One participant explained:

I don’t have television or internet, but I sometimes listen to the radio, and I heard they announced that there are vaccines. In our community, people didn’t know about this because not everyone listens to the radio, and even those who knew didn’t know exactly how and what. What we did see were many pashkevils that warned about the dangers in the vaccines, so we gave up on them. Many families in our community didn’t get vaccinated because of this (Participant 3).

Indeed, numerous pashkevils circulated in Haredi areas urging residents to avoid COVID-19 vaccination. For example, one pashkevil distributed by the zealot circles (i.e., highly insular Haredi subgroups characterized by strong opposition to state authority and strict adherence to religious norms) stated:

The great Torah sages of Israel oppose the vaccines in light of the facts and reliable testimonies about terrible dangers to body and soul from the COVID-19 vaccines! And hundreds of people have already been harmed, and dozens have died (February 2021, Jerusalem).

Similarly, another pashkevil:

After thorough examination by important rabbis […] it was found that there are concerns in the COVID-19 vaccines of vile plots and terrible severe dangers, God forbid! And there is a great obligation to act and activate as much as possible to prevent this danger and evil from Israel! (March 2021, Beit Shemesh).

As a central intra-sectoral communication tool, pashkevils frequently disseminated unreliable and outdated information, posing tangible risks to public health—a pattern that recurs throughout the findings.

### Rabbinic versus professional authority

Another intra-community channel frequently mentioned by participants was rabbinic guidance. Consistent with Haredi tradition, rabbinic authority extended beyond spiritual matters to secular issues, including health. Participants noted that during the COVID-19 pandemic, particularly in its early waves, many rabbis provided their communities with behavioral guidance. These directives often contradicted MOH guidelines. When exposed to MOH social distancing guidelines, a minority of participants found themselves confused by rabbis’ contradictory instructions, attempting to navigate between religious and professional medical authority to protect their families’ health. One participant described:

Because of the MOH guidelines, I was afraid to send the children to yeshiva, but when I heard that Rabbi Kanievsky [considered at the pandemic’s height to be the leading halachic authority] said it’s forbidden to cancel Torah study, I sent them to study at the yeshiva. After a few days, one of the neighbors got infected and needed an oxygen tank, and then I preferred they stay home (Participant 4).

Another participant recounted similarly:

Following Rabbi Kanievsky’s words, we sent the boys to yeshivas, but my sister-in-law, who is a nurse, told us it’s not advisable. We deliberated a lot. My wife was pregnant, and we were afraid of the risks. On one hand, we did not want our son to miss Torah study; on the other hand, we did not want to disobey the rabbi. In the end, I called a doctor who told us unequivocally to obey the MOH guidelines, and that’s what we did with a heavy heart (Participant 5).

These accounts reveal the complex authority dynamics this participant navigated: a dilemma between obeying rabbinic versus professional authority. Unlike these two participants, however, many participants—particularly from more insular groups—expressed strong adherence to rabbinic authority even when it conflicted with medical recommendations, reporting no hesitation. As one participant explained:

In our Hasidic court, the Rebbe said it’s better we gather together, not in isolation, so we’ll get infected from each other and natural immunity will be created. Therefore, we don’t maintain isolation, but even encourage young people to gather together. I know some doctors claim this isn’t good, but if this is what our Rebbe said, I trust him (Participant 6).

Another participant elaborated:

For a Haredi man, even things that seem illogical—when said by the religious leader of the generation—are not subject to challenge. If Rabbi Kanievsky said Torah study must continue, there is no room for doubt. He has a deep understanding of reality shaped by lifelong Torah study.There’s no place to compare Rabbi Kanievsky to a doctor, even if he’s a big specialist (Participant 7).

In line with this, one pashkevil declared:

Clear Torah Instruction: We were instructed to publicize, following the directive of the Torah sages, Rabbi Shmuel Wosner OBM, and Rabbi Chaim Kanievsky *Shlita* [may he live and be well], it has been instructed that beginning tomorrow […] the study halls should reopen, and Torah study should resume (March 2021, Beit Shemesh).

For many participants, “Clear Torah Instruction” represents the ultimate authority, not to be questioned.

### Religious interpretation versus scientific interpretation

Another major health barrier, related to the Haredi population’s tendency to prioritize religious over medical authority, stemmed from religious interpretations of the pandemic’s meaning that contradicted scientific perspectives on its circumstances and implications. Some participants, especially from extremist groups, viewed the pandemic as divine punishment for modesty transgressions. One participant explained:

This pandemic is a message from God that our women are not modest enough. They walk around in tight clothing and skirts that are too short. Many women dress inappropriately and expose their bodies, and now, because of COVID-19, all women have to cover their faces with masks and be hidden from the public (Participant 8).

Another participant connected the pandemic’s name to modesty violations:

The word Corona [COVID-19 in Hebrew] is from the word crown. A Jewish woman is like a queen who is obligated to wear a crown—head covering. Because many women didn’t adhere to head covering, and even among those who did, hair peeked out —which is a severe transgression—Corona conveys an important message: that women must adhere to covering their heads (Participant 7).

Such interpretations appeared frequently on pashkevils, likely influencing these participants. One pashkevil stated: “You threw away the crown on the woman’s head—the kerchief—and replaced it with a wig, now will come the ‘Corona’—crown. Measure for measure” (March 2020, Jerusalem). In other words, Corona—derived from “crown”—afflicted the Jewish people due to Haredi women’s failure to maintain their “head crown” (kerchief covering), replacing it with wigs considered far less modest in most Haredi circles.

Other modesty-focused pashkevils emphasized violations of gender separation. For example, one stated:

You didn’t maintain separation on buses and stood crowded, men and women together, God forbid, and many girls were making contact with the driver […], and now it’s forbidden to stand on the bus, and they keep distance from the driver […]. You walked in the streets with men and women stuck to each other, standing crowded in all kinds of places; now it’s forbidden for people to get close to each other (April 2021, Beit Shemesh).

Yet another interpretation viewed COVID-19 as a punishment for smartphone and internet use, as one participant explained:

On the internet, on smartphones, people are exposed to abominations. COVID-19 is a result of moral deterioration. The internet is a big stumbling block that many men fail with. Now the punishment is to avoid the possibility altogether of looking at people in the street because it’s forbidden to go outside (Participant 9).

These religious interpretations led many participants to prioritize spiritual over medical responses. Rather than viewing social distancing, mask-wearing, and vaccines as primary interventions, many emphasized that “ending the pandemic required strict adherence to women’s modesty regulations rather than compliance with MOH directives” (Participant 10).

Some pashkevils similarly identified modesty improvement as key to eradicating the pandemic. One stated:

Salvation is near at hand—now is not the time to miss this sacred chance. It is the moment to cast aside wigs and form-fitting, revealing clothing, to embrace genuine modesty, and through the virtue of modest women, we will be worthy of full redemption (May 2020, Jerusalem).

Regarding smartphones and the internet, several participants argued that redemption from COVID-19 would occur only if people disconnected from smartphones and the internet. As one explained:

If people stop being exposed to unworthy stuff on their phones and give up the internet, you’ll see that people will get sick less and won’t get infected. It’s proven. In my house, we don’t use the internet except for my oldest son—and he’s the only one who got sick. (Participant 9)

Beyond prohibition adherence, the most prominent belief among participants was that Torah study provides spiritual protection against the virus. Consequently, they vehemently opposed MOH guidelines mandating yeshiva closures. One participant explained: “Torah is the source of life for the Jewish people, and the life system cannot be disconnected even in times of emergency… Closing the yeshiva is like causing death—an action much more dangerous than possible infection” (Participant 10).

Similarly, another participant emphasized: “We always say—Torah protects and saves. Torah study guards and protects the person and the people of Israel. For this reason, it is forbidden to close the yeshiva” (Participant 6). This belief created direct conflict with public health measures requiring educational institution closures, as participants viewed continued Torah study as more important for community protection than adherence to medical guidelines.

## Discussion

This study identified three interconnected barriers that influenced access to healthcare services —particularly preventive services, public health information, and engagement with official health guidelines—and the flow of health information within the Haredi community in Israel during the COVID-19 pandemic, with significant implications for health equity in religious minority populations. Beyond identifying these barriers, the study contributes conceptually by demonstrating that healthcare access in religious minority contexts is governed by legitimacy structures embedded in communication ecologies, rather than by exposure to information alone.

At the same time, it is important to emphasize that these patterns were not uniform across the Haredi population. While the findings highlight mechanisms that may generate resistance to public health measures, participants also reflected varying degrees of engagement with medical guidance, and existing literature points to significant variation between mainstream and more insular groups. This heterogeneity is essential for understanding how communication ecologies operate differently across subgroups within the community.

The first barrier, internal versus external-secular communication, involved reliance on internal channels—word of mouth, internal news hotlines, and pashkevils —that often transmitted partial or misleading information, sometimes conflicting with MOH guidelines. The second barrier, rabbinic versus professional authority, manifested in the consistent prioritization of rabbinic rulings over medical advice, with most participants viewing rabbinic authority as the ultimate source of guidance, offering insights beyond what medicine can provide. The third barrier, religious versus scientific interpretation, reflected views of the pandemic as divine punishment for modesty violations and other sins, leading community members to seek spiritual remedies—such as increased modesty and Torah study—instead of medical interventions.

**Scheme. 1:**
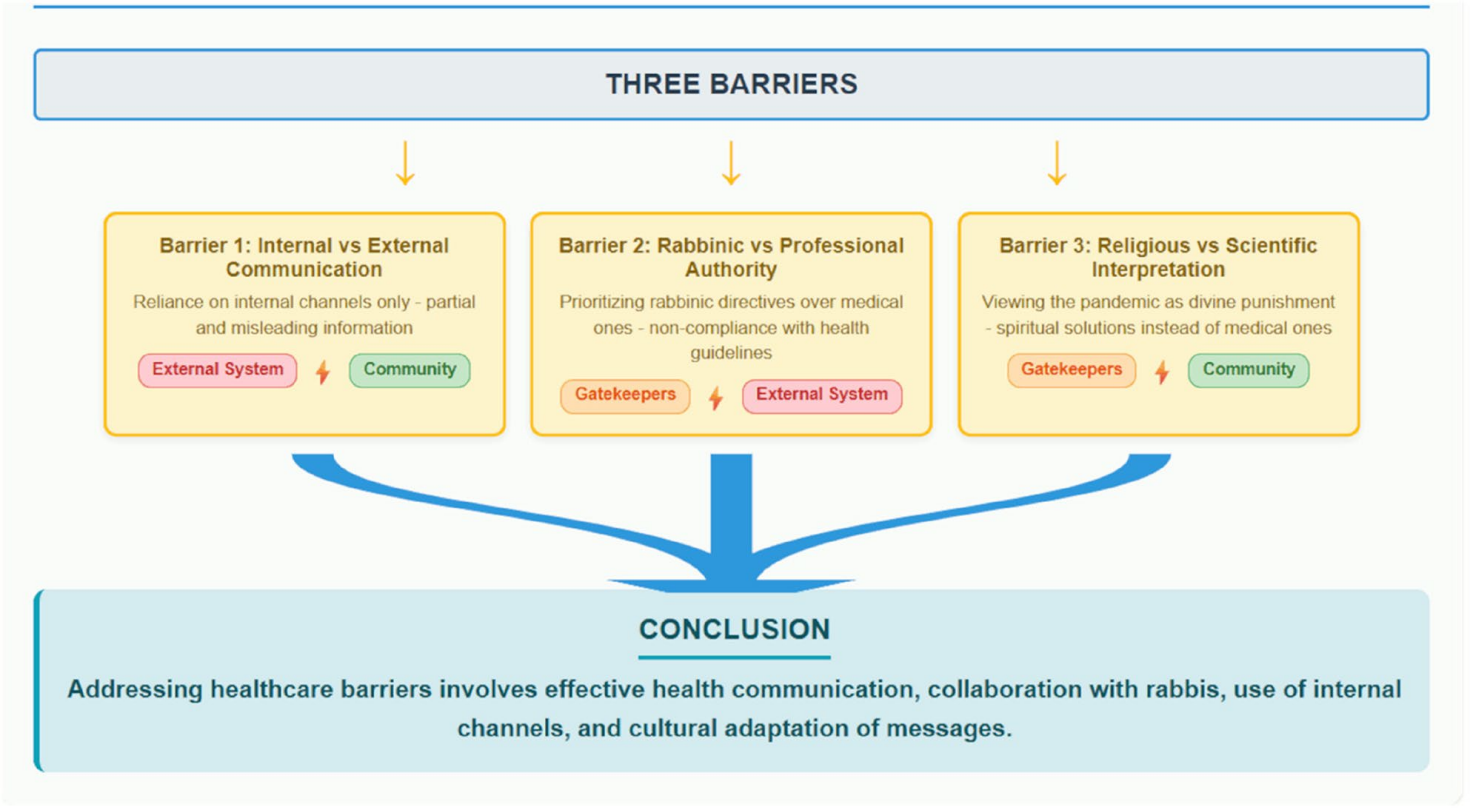
Healthcare barriers in the haredi community during COVID-19: application of the communication ecology theory model

While presented as analytically distinct, these three barriers do not operate independently. Rather, they function as interrelated components of a single bounded communication ecology, in which each element reinforces and sustains the others. Internal communication channels shape exposure to information, rabbinic authority structures determine how that information is interpreted and legitimized, and theological frameworks provide the broader meaning system through which both communication and authority are understood. Together, these layers form a coherent system in which information is not merely transmitted but filtered, reinterpreted, and validated according to internally defined criteria of legitimacy (Scheme 1).

As illustrated in Scheme 1, these three barriers are best understood as mutually reinforcing layers within a single communication ecology rather than as separate factors. The model highlights how information flows through internal channels, is filtered through authority structures, and is ultimately interpreted within a theological framework, producing a closed system of meaning and legitimacy. This integrated perspective deepens the analysis by showing how healthcare barriers are constructed through the interaction between communication, authority, and meaning-making processes.

The communication ecology framework proves particularly valuable in interpreting these findings, as it moves beyond individualistic models of health behavior to examine how information flows through culturally structured networks [44]. Recent studies have further extended Communication Ecology Theory in the context of public health and crisis communication, emphasizing the dynamic role of communication environments and trust networks in shaping responses to health emergencies [58, 59].

Within the Haredi community, the communication ecology operates as a closed system as much as possible, with clear boundaries that filter, reinterpret, and sometimes block external health messages. This insulation reflects a deliberate cultural strategy to preserve religious authenticity and protect against perceived secular contamination [22].

Participants’ descriptions of receiving only fragmented updates about the pandemic through internal news hotlines, or of learning about the crisis from neighbors rather than official sources, expose the consequences of media isolation. Similar patterns appear among religious minorities worldwide, including Orthodox Christian and Muslim communities in Italy [5] and the Amish in the United States [19], where closed communication systems increase exposure to misinformation.

One significant aspect of internal communication was the dissemination of anti-vaccine messages, warnings about vaccine “plots,” and claims about vaccine-related deaths. This vaccine hesitancy, characterizing primarily extremist Haredi groups, extends beyond COVID-19 to other vaccines [60–62], and reflects patterns documented among religious minorities worldwide [63, 64], influenced by institutional mistrust and both structural and ideological barriers [41, 61, 65].

However, vaccine hesitancy does not characterize the entire Haredi population; notably, the majority were vaccinated during COVID-19 [66] and routinely vaccinate their children [62]. This underscores the importance of culturally tailored, authority-mediated communication, where legitimacy builds through alignment with community values and endorsement by respected leaders [41].

The findings regarding rabbinic and professional authority highlight a fundamental tension in how secular health systems communicate with religious communities [3, 67]. In many Haredi segments, rabbinic guidance extends beyond religious law to multiple areas of life [22], including COVID-19 rulings on school closures, synagogue attendance, and vaccination [68]. Participants who heard both MOH guidelines and contradictory rabbinic instructions experienced genuine conflict between protecting family health and honoring religious obligations. For most, however, rabbinic authority was non-negotiable.

This dynamic aligns with broader research showing how clergy serve as “moral interpreters” of medical guidance, determining which interventions align with religious law [4, 5]. When religious leaders perceive health policies as imposing secular values, they may frame compliance with health policy as a spiritual compromise [3].

Not all Haredi leaders sanctioned violations of MOH guidelines [34, 69]. Rabbinic authority is functions as a gatekeeper mechanism [40]: when public health officials communicate respectfully, translate biomedical reasoning into religious language, and show cultural humility, cooperation becomes possible [14]. Effective health communication in insular religious communities thus requires shifting from a confrontational approach—where secular authorities issue unilateral directives—to a collaborative model that views rabbinic leaders as key mediators. This aligns with principles of cultural competence in public health, which emphasize co-creating interventions with community leaders rather than imposing external frameworks [11, 70].

The findings regarding religious interpretations of the pandemic reflect bigger cosmological differences between religious and secular contexts regarding disease causation and treatment [5, 71, 72]. Importantly, these religious interpretations were not uniformly distributed across the Haredi population. Both the interview data and existing literature suggest variation between more mainstream Haredi groups and more insular or extremist factions. While some participants—particularly those affiliated with more conservative communities—framed COVID-19 in strongly theological terms, others demonstrated greater engagement with medical guidance and public health measures.

In particular, more mainstream segments of the community demonstrated greater openness to medical guidance and, in some cases, cooperation with public health directives, especially when such guidance was mediated through trusted rabbinic authorities. This variation underscores that resistance should not be understood as a uniform or defining characteristic, but as one possible outcome within a broader and internally differentiated communication ecology.

Similarly, the pashkevils analyzed in this study should not be interpreted as representing the full spectrum of Haredi opinion. Rather, they often reflect more polemical or ideologically driven messaging that may be more prevalent in certain subgroups. As such, they provide insight into circulating discourses within the communication ecology, but not necessarily into majority views or practices.

Some participants interpreted COVID-19 as divine punishment for modesty violations, mixed-gender interactions, and secular media exposure. Placing collective responsibility on women’s clothing is a recurring theme in Jewish history 73, 74, 75, 76, 77, 78], including early 20th-century attributions of exile to modesty violations [79].

The emphasis on women’s modesty violations as a primary cause of divine punishment reflects deeply embedded patriarchal structures, where women’s bodies are constructed as sources of both communal purity and impurity [80–84], while attributing the pandemic to smartphone use reflects the perception of technological intrusion as a spiritual threat [85, 86].

One theological concept with particular implications is the belief that Torah protects and saves (Torah megina umetsila)—the conviction that Torah study provides metaphysical protection from harm. Participants explained through this belief the importance of keeping yeshivas open, arguing that stopping study constitutes a greater spiritual danger than exposure to the virus. This is not irrational but rational within a different epistemic system, where spiritual causation is as real as biological causation.

Such interpretations have significant behavioral implications. If disease is divine retribution, the appropriate response is repentance and increased religious observance rather than medical interventions, which explains why participants prioritized Torah study over vaccination or social distancing. This pattern is not unique to the Haredi community. It reflects the process by which religious communities evaluate health guidelines according to whether they align with or violate core theological principles [18].

Across all three barriers identified in this study—communication systems, authority structures, and theological worldviews—trust emerges as the fundamental thread. The Haredi community’s healthcare barriers during COVID-19 were not simply about access to information or medical services, but fundamentally about where trust is placed and how it is earned—an insight that is central to advancing health equity in culturally and religiously diverse societies. Trust in internal communication channels over external media, trust in rabbinic authority over medical expertise, and trust in theological explanations over scientific ones all reflect a coherent system in which legitimacy flows from religious sources rather than secular institutions. Taken together, these findings suggest that healthcare barriers in this context are not additive but systemic: the interaction between communication channels, authority structures, and theological meaning-making produces a self-reinforcing ecology that shapes both access to information and its interpretation.

This pattern reflects the community’s negotiation of epistemic sovereignty—the right to determine who may define truth, interpret danger, and prescribe remedies. The Haredi community’s bounded communication ecology preserves religious identity, cohesion, and interpretive autonomy [86]. What secular authorities perceive as “non-compliance” is, from within the community, a rational response within a different epistemic system.

This study advances Communication Ecology Theory by demonstrating how religious authority structures function as legitimacy filters within bounded communication systems. While previous applications emphasized technological and institutional layers [44], this research reveals how theological worldviews constitute a distinct ecological layer that fundamentally shapes information flow and trust in religious minority communities. In doing so, it extends the theory beyond descriptive mapping of communication networks toward an explanation of how epistemic authority and cultural meaning systems actively produce inequalities in access to healthcare.

While this study focused on the Haredi community in Israel, it is important to situate its findings within the broader Israeli context. Similar dynamics of negotiating trust, authority, and health communication have been observed among other minority groups in Israel, including Muslim Arab communities, which navigate complex relationships between community norms and state institutions [87]. Although these groups differ in important cultural and structural ways, this comparison highlights how bounded communication environments and trust dynamics may shape health behavior across diverse minority contexts. Future research may further explore these parallels to deepen understanding of health communication within multi-ethnic societies.

These insights extend beyond Israel to other minority groups and conservative religious communities worldwide [1, 2, 5]. Whether examining Evangelical responses in the United States [17], or Muslim communities in Europe [88], external health directives are filtered based on their alignment with religious identity and authority.

The implications extend beyond immediate crisis response. These patterns persist beyond COVID-19 in measles outbreaks [89], HPV vaccine resistance [90], and influenza vaccine hesitancy [91]. Similar barriers are likely to emerge in future epidemics unless coordinated strategies are developed that address the complex interaction between public health institutions, community leadership, social norms, and levels of trust. Taken together, these findings suggest that such changes must recognize that barriers to healthcare in insular religious communities are fundamentally communicative rather than merely structural. Health messages acquire legitimacy only when mediated through trusted authority structures and aligned with culturally embedded epistemologies. Consequently, effective health communication must move beyond unidirectional information transfer toward culturally grounded collaboration that recognizes internal communication ecologies, engages religious leadership, and addresses structural inequities. Strengthening mutual trust and cultural competence is therefore essential not only for crisis response but also for long-term public health resilience across diverse minority populations.

### Limitations and future research

This study has several limitations. As with any qualitative research, sample bias may exist, as only those willing to participate were interviewed. In addition, the sample excluded participants under 25 and single individuals, which may limit the understanding of younger and unmarried community members’ perspectives. The small sample size also prevented representativeness and generalization, as well as quantitative analysis of sociodemographic variables and sub-sectoral affiliations. Consequently, the findings may not fully reflect the diversity within the broader Haredi population.

Another limitation concerns the scope of perspectives represented. The study explored barriers to healthcare based on interviews with Haredi individuals and analysis of pashkevils, reflecting only one side of the communication dynamic. The perspective of the MOH and other healthcare providers was not examined. In addition, although participants described their primary sources of information, the study did not systematically examine additional external sources such as community clinics, local healthcare professionals, or informal interactions with medical staff. As a result, the findings may not fully capture the range of information to which participants were exposed. The study included pashkevils from certain locations, which potentially overlook pashkevils in other areas expressing different discourses. In addition, pashkevils mostly reflect the early phase of the pandemic, and later ones may have conveyed different emphases.

In light of these limitations, future research should expand both methodological and comparative scope. Future studies could, in the post-pandemic context, use quantitative methods to assess healthcare access, trust in medical institutions, and health behaviors, allowing statistical analysis of variations across age, gender, education, and sub-sectoral affiliation. Longitudinal research would clarify how trust, health attitudes, and attitudes evolve over time—particularly as integration with broader Israeli society increases—and whether the barriers persist or diminish.

Including the MOH’s perspective would provide a more comprehensive understanding of bidirectional communication challenges. Future research should also systematically examine additional external sources of health information, such as community clinics, local healthcare professionals, and informal interactions with medical staff, in order to provide a more comprehensive understanding of the communication environments shaping health behavior in Haredi communities.

Comparative research involving multiple religious minority groups—both within Israel and internationally—could identify which barriers are community-specific versus universal. Cross-national comparisons between Haredi communities in Israel, the United States, and Europe may reveal how national health systems shape trust and compliance among religious minorities.

Given recent measles outbreaks, research on vaccine hesitancy beyond COVID-19 remains relevant. Finally, intervention studies testing culturally tailored health communication strategies—designed in collaboration with religious leaders—could offer evidence-based models for future public health crises.

### Implications for public policy

Effective public health engagement in culturally insular communities requires more than the dissemination of accurate biomedical information. Healthcare disparities in religious minority communities often reflect legitimacy gaps rather than mere information deficits, underscoring that advancing health equity requires engagement with trusted internal authority structures. Public health strategies should therefore be designed to operate through trusted internal authority structures—particularly rabbinic leadership and established community communication channels—rather than relying exclusively on external state media.

Collaborative frameworks between public health institutions and religious authorities should be institutionalized before crises, including joint advisory forums, culturally competent mediators, and pre-approved emergency communication protocols. Establishing long-term trust infrastructures is essential for reducing resistance and improving responsiveness during future public health emergencies.

Such collaboration can be operationalized through concrete mechanisms, including the establishment of joint advisory committees composed of public health officials and recognized rabbinic leaders, the use of culturally embedded mediators to translate health guidelines into religiously meaningful terms, and the dissemination of health messages through trusted internal communication channels such as community hotlines and pashkevils. For example, vaccination campaigns or emergency directives could be introduced through pre-coordinated endorsements by rabbinic authorities, accompanied by messaging framed in alignment with religious values such as the preservation of life (pikuach nefesh). These mechanisms illustrate how public health interventions can be integrated into existing communication ecologies rather than imposed externally.

Furthermore, health messaging should be framed to align with public health directives and core religious values. For example, vaccination campaigns and temporary institutional closures can be framed as expressions of communal responsibility, preservation of life (pikuach nefesh), and protection of vulnerable populations—values deeply embedded in religious tradition.

Crucially, public health interventions must operate within existing communication ecologies rather than bypass them. Attempts to circumvent internal authority structures may inadvertently reinforce resistance and deepen mistrust.

Although this study focuses on the Israeli Haredi community, similar authority-centered communication ecologies exist among religious minorities worldwide. The mechanisms identified here—authority mediation, theological framing, and bounded information flows—are therefore likely transferable to other contexts in which religious identity structures health behavior. Recognizing these patterned dynamics is essential for reducing health inequalities in culturally bounded populations.

## Conclusion

This study demonstrates that healthcare barriers in the Haredi community during COVID-19 were not primarily the result of information deficits or irrational resistance, but rather of bounded communication ecologies in which legitimacy is mediated through religious authority and theological interpretation. Internal communication channels, rabbinic authority structures, and religious meaning-making collectively shaped how public health directives were received, filtered, or resisted.

For public health systems, the key challenge is therefore not merely to improve message clarity, but to engage with the authority structures and communication networks that define trust within culturally insular communities. Public health interventions must operate within existing communication ecologies rather than bypass them.

Although this study focuses on the Israeli Haredi case, similar authority-centered communication systems characterize many religious minority communities worldwide. Recognizing how legitimacy, theology, and internal media shape health behavior is essential to designing culturally responsive strategies to reduce health inequalities across diverse populations.

## Supplementary Information

Supplementary Material 1.

## Data Availability

The datasets generated during the current study are not publicly available due to its sensitive nature, but are available from the corresponding author on reasonable request.
