# Supplementary material for: Authority, trust, and healthcare communication in a religious minority: the case of the Haredi community in Israel during COVID-19
Source: BMC Public Health. 2026 Apr 30;26:1990. doi: 10.1186/s12889-026-27338-9 (PMC13321643; doi:10.1186/s12889-026-27338-9)
Supplement: Supplementary file 1 — Supplementary Material 1. [file 12889_2026_27338_MOESM1_ESM.docx]

**Supplementary File S1**

**Semi-Structured Interview Guide:** Healthcare Communication and Decision-Making in the Haredi Community During COVID-19

The interviews were designed to explore healthcare communication processes, authority structures, and religious interpretations of the COVID-19 pandemic within the Haredi community.

Interviews were conducted in Hebrew and adapted flexibly to allow participants to elaborate on issues they considered significant.

*Opening Invitation*

Please tell me about your experience during the COVID-19 pandemic.

[Participants were encouraged to describe events, feelings, challenges, and significant moments in ways they considered meaningful.]

- Can you describe your family situation during the COVID-19 pandemic?
- How did the pandemic affect your daily routine, work, or religious life?
- Did you or members of your family experience COVID-19 infection? If so, how did you respond?

*Information and Communication*

- How did you come to understand what was happening during the pandemic?
- What were your main sources of information during the pandemic?
  - What kinds of information were available to you, and how did you engage with them?
- How did people around you talk about the pandemic?
- Can you describe situations in which information influenced your behavior or decisions?

*Decision-Making and Authority*

- How were health-related decisions made in your household or community during this period?
  - How did you make decisions regarding vaccination, school attendance, synagogue participation, or quarantine?
- Who or what shaped these decisions?
  - What role did rabbis play in guiding decisions related to COVID-19?
- Can you recall moments of uncertainty or deliberation? Please describe them.
- How were different forms of guidance understood or negotiated?
- Did you encounter differences between information from state authorities and community sources? If so, how did you navigate these differences?
  - Did you ever experience tension between rabbinic guidance and medical recommendations?

*Religious and Cultural Interpretations*

- How was the pandemic interpreted within your social or religious environment?
- What meanings, if any, were attributed to the outbreak?
- In what ways did religious life intersect with everyday health practices during this time?

*Healthcare Practices and Challenges*

- Please describe your experiences, or those of people close to you, with seeking medical care during the pandemic.
- What factors made it easier or more difficult to respond to health recommendations?
- How were preventive measures understood and incorporated into daily life?

*Community Context*

- How would you characterize the broader community response to the pandemic?
- Were there differences in how people reacted or behaved?
- What role did communal structures play during this period?

*Reflective Closing*

- What do you think is important for people outside your community—including public health professionals—to understand about communities such as yours in relation to COVID-19 and other health-related issues?
- What could have improved communication between the Ministry of Health and the Haredi community?
- Is there anything else you would like to share that has not been discussed?
